# Association Between RFC1 G80A Polymorphism and Acute Lymphoblastic Leukemia: a Review and Meta-Analysis of 10 Studies

**Published:** 2016-03-15

**Authors:** M Forat-Yazdi, F Hosseini-Biouki, J Salehi, H Neamatzadeh, R Masoumi Dehshiri, Z Sadri, F Ghanizadeh, R Sheikhpour, H Zare-Zardini

**Affiliations:** 1 **Department of Internal Medicine*, *S*hahid Sadoughi University of Medical Sciences, Yazd, Iran.***; 2 ***Department of Psychology,*S*hahid Sadoughi University of Medical Sciences, Yazd, Iran.***; 3 **Faculty of Pharmacy,S*hahid Sadoughi University of Medical Sciences, Yazd, Iran.***; 4 **Hematology and Oncology Research Center*, *S*hahid Sadoughi University of Medical Sciences, Yazd, Iran.***; 5 **Nutrition and Food Security Research Centre, Shahid Sadoughi University of Medical Sciences, Yazd, Iran.**; 6 **Department of nursing, Yazd Brach, Islamic Azad University, Yazd, Iran**

**Keywords:** Acute lymphoblastic leukemia, Genetic polymorphism, G80A Polymorphism, Meta-analysis, RFC1

## Abstract

**Background:**

Evidence indicates RFC1 G80A polymorphism as a risk factor for a number of cancers. Increasing studies have been conducted on the association of RFC1 G80A polymorphism with acute lymphoblastic leukemia (ALL) risk. However, the results were controversial. The aim of the present study was to derive a more precise estimation of the relationship.

**Materials and Method:**

PubMed, Embase, Web of Science, Cochrane database, and Google Scholar were searched to get the genetic association studies between RFC1 G80A polymorphism and ALL. All eligible studies for the period up to February 2016 were identified. Subgroup analyses regarding ethnicity were also implemented. All statistical analyses were done with CMA 2.0.

**Results:**

A total of ten studies comprising of 2,168 ALL cases and 2,693 healthy controls were included in this meta-analysis. Overall, no significant association was detected for allelic model (OR = 1.029, 95 % CI 0.754- 1.405, P=0.000), Dominant model (OR = 1.619, 95 % CI 0.847-3.094, P=0.145), recessive model (OR = 1.169, 95 % CI 10.764-1.790, P=0.429), and homozygote model (OR = 1.288, 95 % CI 0.928-1.788, P=0.130). However, there was an obvious association under the heterozygote model (OR = 1.368, 95 % CI 1.056- 1.772, P=0.018). Also, in the stratified analysis by ethnicity, no significant association of this polymorphism with risk of OC was found in the Asian and Caucasian populations. However, there was not significant heterogeneity between heterozygote genetic model (P = 0.15, I^2^ = 33%) in Caucasian. Therefore, we utilized the fixed-effect model to merge OR value.

**Conclusion:**

Based on the available evidence, no association between RFC1 G80A Polymorphism and ALL risk was observed, even in the subanalysis by ethnicity. The direction of further research should focus not only on the simple relationship of RFC1 G80A Polymorphism and ALL risk, but also on gene–gene and gene-environment interaction.

## Introduction

In general, leukemia is the most common form of childhood cancer, representing about 30% of all childhood cancers (1). Acute lymphoblastic leukemia (ALL) is the most prevalent type, accounting for approximately 80-85% of childhood leukemias, whereas acute myeloid leukemia (AML) represents about 15-20% (2,3). ALL is a neoplasm of immature lymphoid progenitors that is most commonly of B cell lineage. According to the immunophenotype, ALL is first classified into B-cell progenitor ALL (BCP-ALL) and T-cell ALL (T-ALL) and secondly sub-classified according to cytogenetic and molecular genetic abnormalities (3,4).It suggested that ALL is likely to arise from interactions between exogenous or endogenous exposures, genetic susceptibility and chance (5). 

It has been known for several decades that the majority of childhood ALL cases harbour chromosomal abnormalities such as translocations or aneuploidy (6). ALL is a heterogeneous disease with cytogenetic and molecular genetic abnormalities are known in approximately 75% of the cases. Dysregulation of the T-cell receptor (TCR) genes TLX1, TLX3, HOXA, TAL1, TAL2, LMO1, LMO2, and LYL1 often occurs in T-ALL. In BCP-ALL these abnormalities include hyperdiploidy (>50 chromosomes), hypodiploidy (less than 44 chromosomes), and the translocations t(12;21)(p13;q22) encoding ETV6-RUNX1, t(1;19)(q23;p13) encoding TCF3-PBX1, t(9;22)(q34;q11) encoding BCR-ABL1, and MLL rearrangements (7,8). Beside, a minority of all ALL cases is associated with inherited, predisposing genetic syndromes as for example Down’s syndrome (2). Hereditary factors are likely to have a considerable role in the predisposition to childhood leukaemia (9). Recent studies shown that the family history of cancer may be a risk factor for childhood ALL (8,9). The association was particularly clear when restricted to family history of AML. In addition, the inherited variation of some specific genes has been shown to influence the susceptibility of childhood leukaemia (7,8). Reduced folate carrier 1 (RFC1)/solute carrier family 19 members 1 (SLC19A1) gene, located on chromosome 21, encodes a high-capacity, bi-directional transporter of 5-methyl-tetrahydrofolate and thiamine monophosphate (10, 11). In addition, RFC1 actively transports antifolate chemotherapeutic agents into cells (12,13). RFC1 also plays critical role in folate homeostasis of mammalian cells, where it is down regulated in response to folate deficiency (14). 

To date, a variety of molecular epidemiological studies have been conducted to examine the association between RFC1 G80A polymorphism and ALL risk, but the results remain inconclusive. Here, we performed a meta-analysis to derive a more precise evaluation of the association between RFC1 G80A polymorphism and ALL risk.

## Materials and Methods

**Identification and eligibility of relevant studies**

Two authors independently conducted a systematic literature search in the PubMed, Elsevier, and Google scholar databases to identify studies about the relationship between RFC1 G80A polymorphism and ALL risk (up to December 20, 2015). The search terms and keywords used were as follows: “Reduced Folate Carrier 1” or “RFC1 G80A”, ” or “solute carrier family 19 members 1 (SLC19A1)”, “polymorphism” or “variant”, “Rs1051266 “**, **and “acute lymphoblastic leukemia” and “ALL”**. **The search was limited to English language papers. A manual search for references cited in the eligible articles was also performed to look for additional studies. Studies included in our meta-analysis have to meet the following criteria: (1) use a case–control design and (2) sufficient data for examining an odds ratio (OR) with 95% confidence interval (CI). Major reason for exclusion of studies was no control population**.**

**Data extraction**

Two investigators independently extracted data according to the inclusion and exclusion criteria and reached a consensus on all the items. The following data were collected from studies: first author, year of publication, ethnicity, and numbers of genotyped cases and controls. Different ethnic descents were categorized as Caucasian and Asian.

**Statistical analysis**

The strength of association of RFC1 G80A polymorphism with ALL risk was assessed by odds ratios (ORs) with 95% confidence intervals (CIs) under Allelic model (A vs. G), dominant model (AG+AA vs. GG), recessive model (AA vs. AG+GG), homozygote model (GG vs. AA) and the heterozygote model (AG vs. GG). The statistical significance of the summary OR was determined with the Z-test. The *Z*-test was used to determine the significance of combined ORs. The heterogeneity between included studies was evaluated by the Q-test. If *P *> 0.05, indicating that there exists no significant heterogeneity, the fixed-effects model (Mantel-Haenszel) was selected to combine the data, otherwise, the random-effects model (DerSimonian-Laird) was applied. Subgroup analyses were performed according to the cancer type (CRC or gastric) and ethnicity (Asians and Caucasians). Sensitivity analysis was performed to assess the stability of results. Funnel plots were drawn to estimate the potential publication bias, in which the standard error (SE) of log (OR) of each study was plotted against its log (OR). The funnel plot asymmetry was assessed with Egger’s test [28]. Publication bias was assessed with Egger test; P<0.05 was considered statistically significant. Hardy–Weinberg equilibrium (HWE) in the control group was tested using the *χ2*-test for goodness of fit. All the tests were two-sided and *P *< 0.05 was considered as statistically significant. All statistical tests for this meta-analysis were performed with CMA.

## Results

**Study characteristics**

A total of ten case-control studies on the association of RFC1 G80A polymorphism with ALL risk were retrieved based on the inclusion criteria with 2,168 cases and 2,693 controls (15-24). Study characteristics were summarized in Table I. Of the included studies, 6 studies were about Caucasians (15-20) with 1,315 cases and 1,695 controls and 4 studies were about Asians (21-24) with 853 cases and 998 controls. The genetic distributions in controls of three case-control studies (Gast et al., 2007, Zhao et al., 2011, Silva et al., 2013) were out of HWE. 

**Meta-analysis**

The association of the RFC1 G80A polymorphism with ALL risk under different genetic models was shown in Table II. As shown, RFC1 G80A polymorphism was not associated with increased risk of ALL under four main models (A vs. G model, OR = 1.029, 95 % CI 0.754-1.405, P=0.00; Dominant model, OR = 1.619, 95 % CI 0.847-3.094, P=0.145; recessive model, OR = 1.169, 95 % CI 10.764-1.790, P=0.429, and homozygote model, OR = 1.288, 95 % CI 0.928-1.788, P=0.130) (Figure 2a). However, there was an obvious association between RFC1 G80A polymorphism and ALL risk under the heterozygote model (OR = 1.368, 95 % CI 1.056-1.772, P=0.018) (Figure 2b).

**Figure 1.**
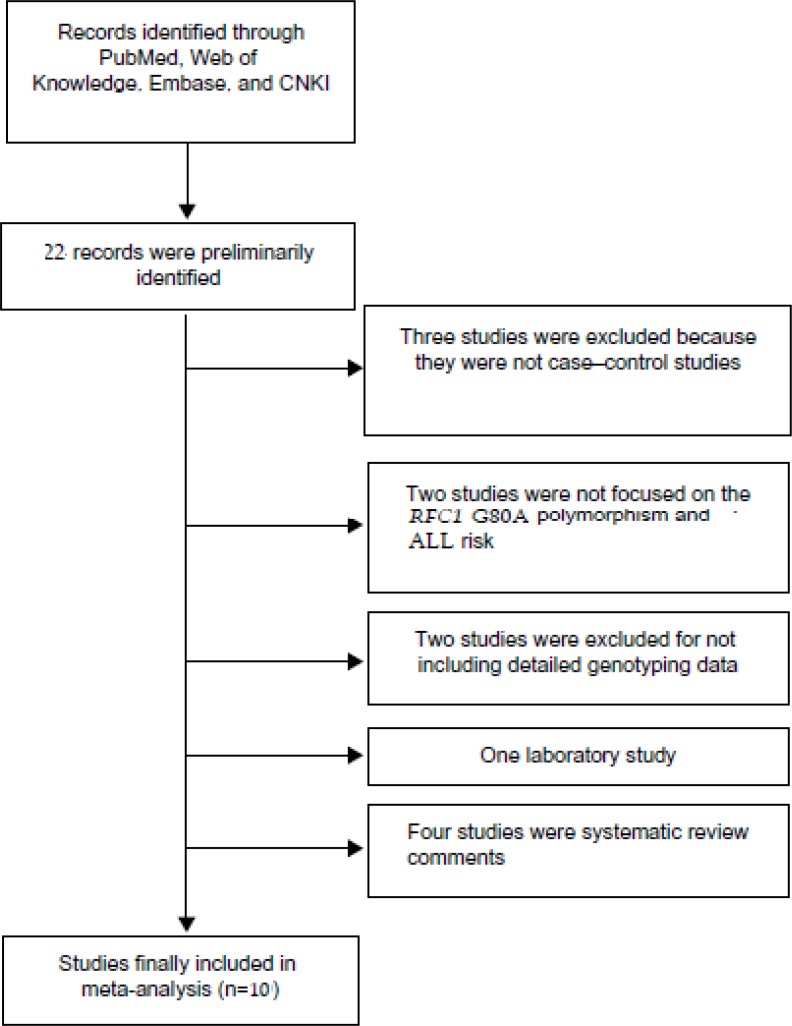
Flow chart of selection of literatures

**Table I:** *The characteristics of the included studies on RFC1 G80A polymorphism acute lymphoblast leukemia risk.*

			**Case**					**Control**						
**Author/Year**	**Ethnicity**	**Case/Control**	**Genotype**			**Allele**		**Genotype**			**Allele**		**HWE**	
			**GG**	**AG**	**AA**	**G**	**A**	**GG**	**AG**	**AA**	**G**	**A**	**P-value**	**X** ^2^
**Whetstine et al., 2001**	Caucasian	54/51	10	24	20	44	60	9	25	17	43	59	0.970	0.001
**Gast et al., 2007**	Caucasian	542/542	125	251	79	596	488	178	256	310	445	639	0.000	62.33
**de Jonge et al., 2009**	Caucasian	241/495	69	120	52	258	224	186	241	68	613	377	0.470	0.519
**Yeoh et al., 2010**	Asian	210/319	62	108	40	232	188	72	170	77	205	433	0.237	1.393
**Chan et al., 2011**	Asian	184/177	43	98	43	184	184	61	75	41	194	160	0.059	3.548
**Metayer et al., 2011**	Caucasian	348/422	106	188	54	399	297	132	205	85	464	380	0.738	0.111
**Yang et al., 2011**	Asian	361/367	93	172	96	357	365	105	191	71	382	352	0.339	0.913
**Zhao et al., 2011**	Asian	98/135	21	53	24	95	101	53	52	30	158	112	0.016	5.762
**Silva et al., 2013**	Caucasian	95/137	21	38	36	80	110	49	56	32	153	121	0.047	3.945
**Karathanasis et al., 2014**	Caucasian	35/48	9	16	10	34	36	15	18	15	35	35	0.083	3.00

**Table II:** *ORs and 95 % CI for RFC1 G80A polymorphism and ALL risk under different genetic models*

**Genetic model**	**OR (95 % CI)**	**P value**	***I*** ^2^ ** (%)**	**P **_heterogeneity_	**Model**
Allele	1.029(0.754-1.405)	0.858	94	0.00	Random
Recessive (AA vs. G-carriers)	1.169(0.764-1.790)	0.429	77	0.000	Random
Dominant (A-carriers vs. GG)	1.619(0.847-3.094)	0.145	94	0.00	Random
AG versus GG	1.368(1.056-1.772)	0.018	66	0.001	Random
AA versus GG	1.288(0.928-1.788)	0.130	70	0.001	Random

**Table III:** *Sub-group analysis of different genetic models by ethnicity (OR, 95 % CI*

**Genetic model**	**OR (95 % CI)**	**P value**	***I*** ^2^ ** (%)**	**P **_heterogeneity_	**Model**
**Caucasian**					
Allele	1.006(0.662-1.530)	0.977	91	0.00	Random
Recessive (AA vs. G-carriers)	1.115(0.851-1.461)	0.188	69	0.001	Random
Dominant (A-carriers vs. GG)	2.161(0.718-6.503)	0.170	96	0.00	Random
AG versus GG	1.303(1.098-1.548)	0.003	0.00	0.868	Fixed
AA versus GG	1.465(0.933-2.302)	0.097	70	0.004	Random
**Asian**					
Allele	0.932(0.513-1.695)	0.818	95	0.00	Random
Recessive (AA vs. G-carriers)	1.077(0.768-1.511)	0.667	54	0.087	Random
Dominant (A-carriers vs. GG)	1.063(0.530-2.131)	0.863	90	0.00	Random
AG versus GG	1.556(0.788-3.073)	0.203	88	0.00	Random
AA versus GG	1.231(0.701-2.162)	0.469	76	0.005	Random

**Figure 2:**
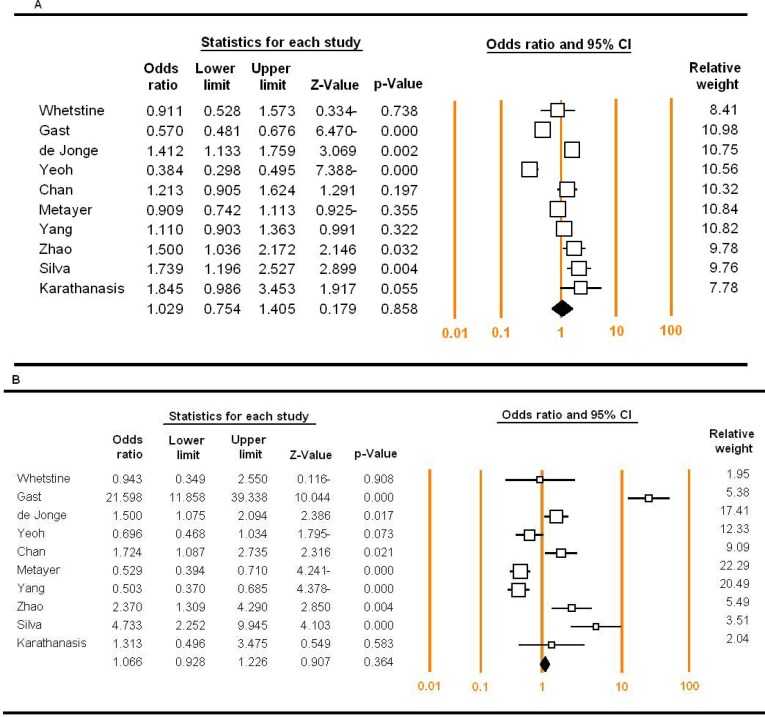
Forest plots for the meta-analysis of the association between RFC1 G80A

polymorphism and ALL risk (**A:** A vs.G and **B:** AA+AG vs. GG).

**Figure 3:**
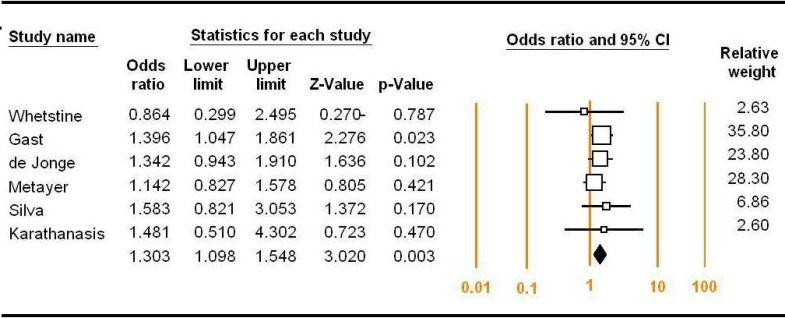
Forest plots for the meta-analysis of the association between RFC1 G80A polymorphism and

ALL risk in Caucasian population under heterozygote genetic model (AG vs. GG, fixed-effects model).

**Figure 4:**
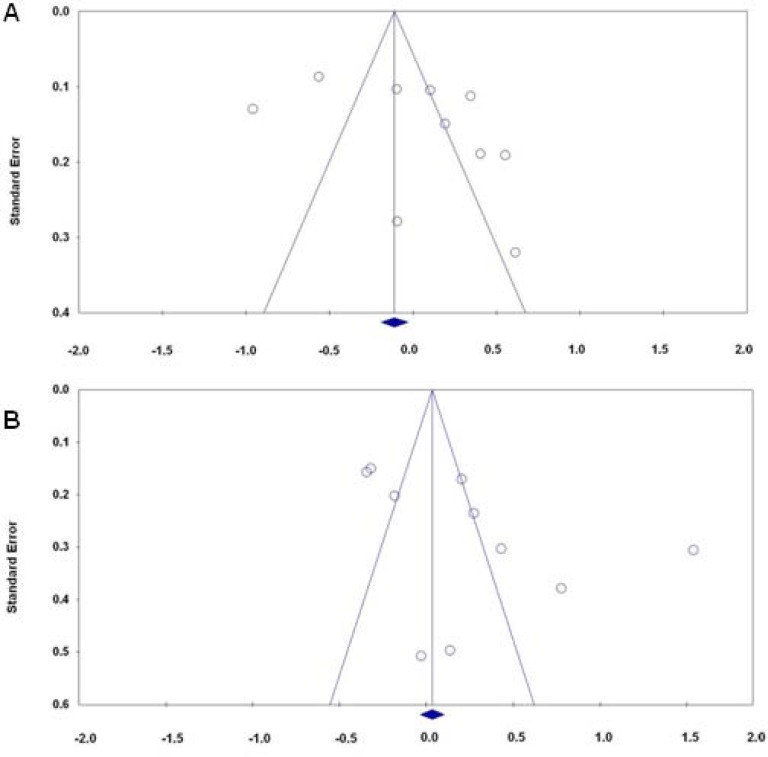
Funnel plot for publication bias in the meta-analysis investigating the association

between RFC1 G80A polymorphism and ALL risk, (**A:** A vs.G and **B:** AA+AG vs. GG).

**Ethnicity Analysis**

Subgroup analyses of the different ethnic groups were performed. There was no evidence of association for Asian subgroup when stratified by ethnicity in all models (A vs. G: OR = 0.932; 95% CI: 0.513-1.695, P = 0.818, AG+AA vs. GG: OR = 1.063; 95% CI: 0.530-2.131, P = 0.863, AA vs. AG+GG: OR = 1.077; 95% CI: 0.768-1.511, P = 0.667, AG vs. GG: OR = 1.556; 95% CI: 0.788-3.073, P = 0.203, AA vs. GG: OR = 1.231; 95% CI: 0.701-2.162, P = 0.469). The meta-analysis results suggested that, in Caucasian, there are not significant differences between G80A polymorphism and ALL risk under four models (A vs. G: OR = 1.006; 95% CI: 0.662-1.530, P = 0.977, AG+AA vs. GG: OR = 2.161; 95% CI: 0.718-6.503, P = 0.170, AA vs. AG+GG: OR = 1.115; 95% CI: 0.851-1.461, P = 0.188, AA vs. GG: OR = 1.465; 95% CI: 0.933-2.302, P = 0.097). However, there was not significant heterogeneity between heterozygote genetic model (P = 0.15, I^2^ = 33%) in Caucasian. Therefore, we utilized the fixed-effect model to merge OR value (Figure 3). 

**Sensitivity analysis**

One single study from the overall pooled analysis was deleted each time to check the influence of the removed data set to the overall ORs. The pooled ORs and 95% CIs were not significantly altered when any part of the study was omitted, which indicated that any single study had little impact on the overall ORs.

**Publication Bias**

Begg’s funnel plot and Egger’s test were performed to assess the publication bias of literatures. The shape of the funnel plots did not reveal any evidence of the obvious asymmetry for all genetic models in the overall meta-analysis (Data not shown). Begg’s test and Egger’s test did not reveal any significant evidence of publication bias for any of the genetic models (A vs. G: P_Begg_ = 0.602 and P_Egger_ = 0.359; AA+AG vs. GG: P_Begg_ = 0.210 and P_Egger_ = 0.082; AA vs. AG+GG: P_Begg_ = 0.210 and P_Egger_ = 0.311; AG vs. GG: P_Begg_ = 0.720 and P_Egger_ = 0.421; AA vs. GG: P_Begg_ = 0.754 and P_Egger_ = 0.514, Figure 4a, 4b).

## Discussion

Acute lymphoblastic leukemia is the most common pediatrics cancer, accounting for 25% of all childhood malignancies. With the present diagnostic techniques the genetic aberrations have found in about 90% of ALL and AML patients. The distribution of the abnormalities In ALL is clearly different in children and adults. Also, the distributions in infants and older children differ remarkably from each other. The differences in frequencies offer a partial explanation to the different outcomes in different age groups.

Several previous studies suggested that RFC1 G80A polymorphism was associated with the risk for ALL. However, other case–control studies reported conflicting results. This may partly be due to a small sample size in each of the published studies and ethnic difference. Meta-analysis is a useful statistical method that combines findings from independent studies.

Meta analysis has great power for elucidating genetic factors in cancer. On the bases of the character of cancer, the effect of one genetic component on the development of the disease can be easily masked by other genetic and environmental factors. A meta-analysis potentially investigates a large number of individuals and can estimate the effect of a genetic factor on the risk of the disease (26). The present study included data from 10 association studies that had investigated the relationship between the RFC1 G80A polymorphism and ALL.

This present meta-analysis, including 20,907 cases and 23,905 controls, concerned the RFC1 G80A polymorphism and ALL risk. In the meta-analysis, we found that the variant genotypes of the RFC1 G80A polymorphism were significantly associated with BC risk. Simultaneously, the same results presented in stratified analysis by ethnicity. We found that the variant genotype of the RFC1 G80A polymorphism, in Asian populations, was associated with significant increase in ALL risk. Although the RFC1 gene production assigned in DNA replication, there was no a significant association of this polymorphism with ALL risk in overall (except heterozygote model), Caucasian (except heterozygote model under fixed-effects model) and Asian populations. Therefore, it seems that the influence of the RFC1 G80A polymorphism may be masked by the presence of other unidentified causal genes involved in ALL. 

In this meta analysis two significant issues addressed, that is, heterogeneity and publication bias, which may influence the results of meta-analysis. There was not a significant publication bias in this meta-analysis, suggesting the reliability of the results. In this meta-analysis, heterogeneity was observed between publications for RFC1 G80A polymorphism in total population group (Table III). The source of heterogeneity may arise from many aspects, such as, ethnicity, country, the sample size of the case and the control group, publication year, genotyping method and so on. In order to explain the main reasons for the heterogeneity across studies stratified analyses by ethnicity was performed. When subgroup analyses were carried out according to ethnicity, this heterogeneity was greatly reduced or removed in some subgroups. The results showed that there was not a significant heterogeneity in the Caucasian only under heterozygote genetic model, implying different effects on ethnic populations.

To the best of our knowledge, this is the first and the most comprehensive meta-analysis undertaken so far for quantitative analyses between RFC1 G80A polymorphism and risk of ALL. However, in interpreting the results, there were some limitations in this meta analysis which should be acknowledged. First, the calculated odds ratios in the present meta-analysis were necessarily crude unadjusted odds ratios, as information about potential confounders, especially environmental exposure patterns, were rarely found in the individual studies. Second, all recruited case–control studies were from Asians and Caucasians, there was no data on the African population in this meta-analysis. Thus, the results may only be suitable for Asians and Caucasians ethnicity. Third, only published studies were eligible in this meta-analysis; therefore, some relevant unpublished studies were inevitably missed, which may lead to bias. Fourth, due to the lack of sufficient and uniform information in original case-control studies, data were not stratified by other factors such as sample sources, genotyping methods, cases gender and age, and so on.

## Conclusion

This meta-analysis suggests the RFC1 G80A polymorphism represents a low risk factor for ALL, especially in Asians. In the future, more studies with large sample sizes should be carried out to clarify the association between RFC1 G80A polymorphism and ALL risk, especially unidentified causal genes and gene–environment interactions.

## Conflict of interest

The Authors have no conflict of interest.
